# Childhood Trauma and Malevolent Creativity in Chinese College Students: Moderated Mediation by Psychological Resilience and Aggression

**DOI:** 10.3390/jintelligence10040097

**Published:** 2022-11-07

**Authors:** Wenfu Li, Linghui Zhang, Zhilei Qin, Jingting Chen, Chuanxin Liu

**Affiliations:** 1 School of Mental Health, Jining Medical University, Jining 272067, China; 2 Department of Child and Adolescent Psychiatry, Shandong Daizhuang Hospital, Jining 272051, China; 3 Labour Union, Jining Medical University, Jining 272067, China

**Keywords:** childhood trauma, malevolent creativity, aggression, psychological resilience, college students

## Abstract

Although a previous study has shown that childhood trauma influences malevolent creativity, aggression and psychological resilience have been linked with childhood trauma and creativity. However, little is known about the complex correlations among these factors in Chinese college students. The present study aimed to investigate the mediating role of aggression and the moderating role of psychological resilience between childhood trauma and malevolent creativity. A total of 389 undergraduates were enrolled in this cross-sectional study. The moderated mediation model was conducted to explore whether aggression mediated the correlation between childhood trauma and malevolent creativity and whether psychological resilience moderated the indirect role of childhood trauma. The results showed that childhood trauma positively correlated with aggression and malevolent creativity and was negatively associated with psychological resilience. Aggression partly mediated the association of childhood trauma with malevolent creativity. Resilience moderated the indirect effect of the mediation model, such that the indirect effect of childhood trauma on malevolent creativity through aggression increased as the level of resilience increased. The study indicated that childhood trauma exposure is associated with malevolent creativity behavior, and aggression mediated this association. The level of psychological resilience differentiates the indirect paths of childhood trauma on malevolent creativity. These results have important implications for preventing and containing expressions of malevolent creativity.

## 1. Introduction

Creativity, as a crucial force for human survival and social progress (Runco 2004), is widely defined as the ability to generate ideas, solutions, or products that are both novel and useful (Runco and Jaeger 2012; Sternberg and Lubart 1999). Creativity is commonly considered to be a benevolent thing. However, recent reports have begun to acknowledge the dark side of creativity, or malevolent creativity generated to purposely harm others (Cropley et al. 2008; Harris and Reiter-Palmon 2015). A wide variety of malevolent creativity instances can be found everywhere. Extreme examples can be creative terrorist attacks or criminal behaviors, and more common instances can be creative deception, theft, cheating, kidnap, or sexual harassment (Cropley and Cropley 2011; Gill et al. 2013; Harris and Reiter-Palmon 2015). These malevolent creativity behaviors usually cause damage in original or innovative ways and therefore are hard to detect and prevent (Gutworth et al. 2018). The academic research identifying predictors of malevolent creativity behavior and interaction mechanisms not only contributes to the systematized understanding of the nature of creativity but also reminds the public that creativity generated to purposely harm others may cause vast hurt to individuals and great damage to the whole society (Jia et al. 2020). Therefore, an in-depth study of the occurrence mechanism underlying malevolent creativity contributes to preventing malevolent creativity behavior and has significant social value.

Malevolent creativity has previously been linked to both environmental and individual factors (Cropley et al. 2014; Gong and Liu 2016; Harris et al. 2013; James et al. 1999; Jia et al. 2020; Perchtold-Stefan et al. 2021). For example, James et al. (1999) pointed out that malevolent creativity is related to a negative social climate. Other studies found that an unfair environment (Clark and James 1999) and social threat (Baas et al. 2019) could increase the likelihood of malevolent creativity behaviors. Additionally, researchers also found a close association between malevolent creativity behaviors and individual personality or personal characteristics. For instance, Jonason et al. (2017) found that Machiavellianism and psychopathy are positively related to malevolent creativity among both males and females, with male-specific associations in psychopathy. In addition, numerous studies indicate that malevolent creativity has been associated with aggression (Harris and Reiter-Palmon 2015), antagonism (Perchtold-Stefan et al. 2021), and integrity (Beaussart et al. 2013). Besides environment and personality factors, other emotional or motivational factors, such as negative emotion (James et al. 1999), approach motivation (Hao et al. 2020), and moral reasoning (Zhao et al. 2022a) could also increase the likelihood of malevolent creativity behaviors. In addition, early adverse life factors are shown to be correlated with the increased emergence of malevolent creativity behaviors (Jia et al. 2020), but the underlying mechanisms accounting for this correlation are largely unknown. In the present study, we aimed to investigate the environmental and individual difference factors shown to relate to malevolent creativity behaviors in the general population.

### 1.1. Childhood Trauma and Malevolent Creativity

Childhood trauma is usually characterized by physical, emotional, sexual abuse and/or physical and emotional neglect, which happened before 18 years old (Bernstein et al. 2003). Previous studies have shown that childhood trauma is correlated with various negative outcomes, such as post-traumatic stress disorder (Doba et al. 2022), food addiction (Wattick and Olfert 2022), schizotypy (Gong et al. 2019), and decreased level of executive function (Bernardes et al. 2020), cognitive flexibility (Spann et al. 2012), working memory (Chiasson et al. 2021), and openness to experience (Fletcher and Schurer 2017) in adolescence and young adulthood. These negative influences usually endure through adolescence and adulthood (Odgers et al. 2008).

Some research studies have shown that the adverse experiences that happened in early life impose limits on benevolent creativity (Zhang et al. 2018). As for the malevolent creativity phenomenon, to date, there has been only one study that has explored the correlation of childhood trauma with malevolent creativity behaviors. Jia et al. (2020) first explored the association between childhood neglect and malevolent creativity in undergraduates. The results indicated that childhood neglect was positively associated with individual malevolent creativity behaviors. As an initial attempt, this research does provide important insight into the effect of childhood adverse experiences on malevolent creativity behaviors, but the underlying environmental mechanisms accounting for this correlation are largely unknown. Basically, the association between childhood trauma and malevolent creativity is demonstrated, but the psychological mechanism still needs to be investigated deeply. Of note, it remains unclear to date that the psychological mechanism that could account for this association (i.e., the mediation effects) and alter it (i.e., the moderation effects).

Numerous studies have shown that childhood trauma, aggressive behaviors, and psychological resilience are associated with malevolent or benevolent creativity (Anser et al. 2022; Harris and Reiter-Palmon 2015; Lee and Dow 2011; Marwa and Milner 2013; Zhang et al. 2018). Therefore, the present research utilized a Chinese adolescent sample to investigate the roles of aggression and psychological resilience in the association between childhood trauma and malevolent creativity. Specifically, we aimed to examine whether aggression mediated the association between childhood trauma and malevolent creativity and whether psychological resilience moderated this mediating process.

### 1.2. Aggression as a Mediator

Aggressive behavior is correlated with various negative outcomes, such as low academic performance, emotional recognition, and social competence (Acland et al. 2021; Chen et al. 2010; Vuoksimaa et al. 2021). In some ways, malevolent creativity can be regarded as aggressive creativity because it is intentional and harmful in nature (Harris and Reiter-Palmon 2015). General Aggression Model (GAM) suggests that those higher in aggression-prone have a natural tendency to think, believe, and perceive in a malevolently-jaundiced way and have a biased tendency to show aggressive responses (Anderson and Bushman 2002). An empirical study found that the trait of physical aggression was positively associated with malevolent creativity measured by divergent thinking tasks (Lee and Dow 2011). Harris and Reiter-Palmon (2015) have also reported that malevolently creative ideas generated in problem-solving tasks were significantly higher in participants who are more implicitly aggressive than in participants who are less implicitly aggressive. Therefore, aggression-prone individuals tend to construct their inner worlds in hostile and competitive ways and spend a huge amount of time and effort generating many different types of aggressive behaviors (Harris and Reiter-Palmon 2015). Each of the aggressive individuals is an expert in thinking aggressively and flexibly when producing harmful behaviors under certain circumstances. This flexibility may increase the likelihood of generating more original and harmful responses or more malevolent creative behaviors (Harris and Reiter-Palmon 2015). Thus, we hypothesize that aggression will positively predict malevolent creativity behavior.

Among various factors that influence a person’s aggression, childhood trauma is widely considered one of the most important factors (Bland et al. 2018; Rasche et al. 2016). Aggressive behavior is one of the externalizing symptoms of individuals with exposure to childhood adversity (Fava et al. 2019). Previous empirical studies have consistently indicated that maltreated individuals are more likely to generate aggressive behavior (He and Xiang 2021; Ma et al. 2022; Schwarzer et al. 2021; Xiao et al. 2021). The association between childhood trauma and aggressive behavior is consistent with the theoretical perspectives of GAM (Anderson and Bushman 2002), which propose that individuals with childhood trauma experience tend to normalize the use of violence and shape it into aggressive scripts. These behavioral scripts will further affect the preparedness for aggressive behavior. Additionally, the longitudinal study also has shown that childhood neglect positively predicts later aggressive behavior, supporting that neglect damages mental functioning obviously (Logan-Greene and Semanchin Jones 2015). The Developmental Traumatology Model (DTM) posits that exposure to childhood trauma increases the risk of post-traumatic stress disorder (PTSD), which is characterized by avoidance, overactive, and mistrust of others (De Beilis and Putnam 1994). PTSD patients are inclined to think that others will deliberately hurt them and thus become more hostile and aggressive (Lawrence-Wood et al. 2021). Considering the influence of childhood trauma on aggression and the association between aggression and malevolent creativity, we can reasonably assume that aggression may be conducted as a mediating variable in the hypothesized connection between childhood trauma and individual malevolent creativity.

### 1.3. Psychological Resilience as a Moderator

Psychological resilience, as one of the crucial protective factors, is defined as the ability to adapt positively to stressors or adversity and keep mental health in the presence of stressful events in the opinion of positive psychology (Kalisch et al. 2015; VanMeter and Cicchetti 2020). Despite exposure to adverse experience in early-life, individuals with higher resilience may not suffer emotional or psychological issues. A growing line of studies suggests that high psychological resilience is positively associated with decreased mental health issues (Anyan and Hjemdal 2016; Min et al. 2015). A longitudinal study also found that about one-third of the high-risk children who had experienced severe stressful life events grew into healthy adults without grievous mental disorders (Werner 1996). Other studies have explored psychological resilience as the moderating variable between childhood trauma and aggression and also as a protective factor against aggressive behaviors (Kim et al. 2015; Nooripour et al. 2022). For instance, Kim et al. (2015) found that psychological resilience could attenuate aggressive behaviors in individuals who had been exposed to early life stress. Nooripour et al. (2022) supported that resilient individuals exhibited fewer aggressive behaviors and had better mental health. Therefore, psychological resilience may play a moderating role in the correlation between childhood trauma and aggressive behaviors. However, relatively little research has touched on the interaction effect of childhood trauma and psychological resilience on aggression.

Previous studies have pointed out that psychological resilience enables people to acquire the positive side of adversity, get rid of negative emotional experiences, and adapt to stressful environments (Tugade and Fredrickson 2004). Other studies also indicated that psychological resilience could alleviate negative emotions and reduce problem behavior in individuals who had experienced childhood trauma (Canale et al. 2019; Chang et al. 2021; Fedina et al. 2021). Additionally, The Rutter’s Model of Development states that psychological resilience could reduce the negative influences of risk factors and minimize the severe adverse reactions to stressful events (Rutter 1999). Based on the literature above, it is reasonable to expect that psychological resilience may also attenuate the possible effects of childhood trauma on malevolent creativity. Therefore, we hypothesized that psychological resilience might moderate the indirect association between childhood trauma and malevolent creativity.

### 1.4. The Present Study

**Figure 1:**
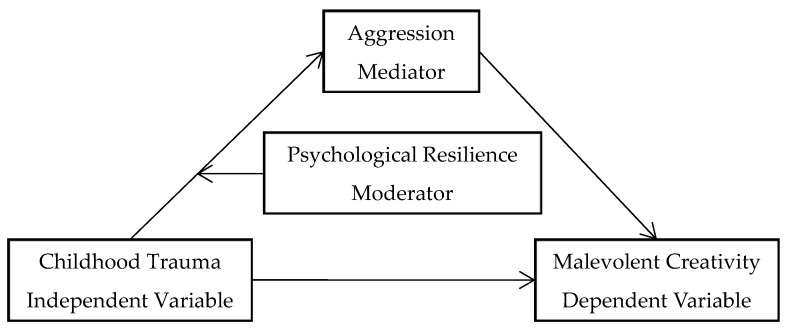
The hypothetical moderated mediation model.

Taken together, the aims of the present study were two-fold. Firstly, the present research investigated whether aggression would mediate the association between childhood trauma and malevolent creativity. Secondly, we explored whether psychological resilience would moderate the correlation between childhood trauma and aggression. Specifically, a structural equation modeling was used to construct a moderated mediation model (Figure 1) to examine the association between childhood trauma and malevolent creativity. Based on the above literature, the present study predicted that childhood trauma would be positively correlated with malevolent creativity (Hypothesis 1). In addition, we predicted that aggression would mediate the association between childhood trauma and malevolent creativity (Hypothesis 2). We also predicted that psychological resilience would moderate the association between childhood trauma and aggression (Hypothesis 3).

## 2. Methods

### 2.1. Participants

The present study recruited 440 Chinese undergraduates. All subjects volunteered for an online survey on the website www.wjx.cn (accessed on 29 March 2022). The questionnaires selected in the present study were all modified or developed following a standard procedure, and the items were all in a concise and easily understandable Chinese version. The survey takes about eight minutes to fill out all questions. The data from 29 questionnaires were excluded from the further analysis because they included invalid answers or consumed time beyond three standard deviations (±3σ). Additionally, the data from 22 questionnaires that included multivariate outliers (beyond ±3σ) were also excluded. Lastly, the valid data included 389 participants, which consisted of 144 males and 248 females, the mean age was 20.53 ± 1.70 years, ranging from 17 to 29 years. Participants were heterogeneous with regard to only-child (only-child = 35.40%, *n* = 136; non-only-child = 64.60%, *n* = 253) and place of birth (city = 25.60%, *n* = 100; town = 23.90%, *n* = 94; country = 50.50%, *n* = 195). G*Power was used to conduct the power analysis with statistical power = 0.95, significance level = 0.05 (two-tailed), and the number of predictors = 7, and the results revealed that the minimum sample of 153 participants would be adequate for detecting a medium effect size of 0.15 for the linear multiple regression analysis followed Cohen (1992). This indicated that the sample size of the present study was appropriate. The research procedure was approved by the local Ethics Committee.

### 2.2. Measures

#### 2.2.1. Short Form of Childhood Trauma Questionnaire (CTQ-SF)

The original CTQ-SF, one of the widely used instruments (Georgieva et al. 2021), was developed to provide a reliable and valid retrospective evaluation of child abuse and neglect (Bernstein et al. 2003). The Chinese version of CTQ-SF was revised by Zhao et al. (2005), which had satisfactory reliability and validity. The scale consisted of 25 clinical items and three validity items. Each item asked about objective experiences and subjective evaluations in childhood and adolescence and was graded on a five-point Likert scale. Participants were asked to choose one of five options ranging from Never to Always. CTQ-SF consisted of five clinical factors: physical abuse, emotional abuse, sexual abuse, physical neglect, and emotional neglect. The examples of items and conceptions of abuse and neglect could be referred to in previous research (Bernstein et al. 1994; Bernstein et al. 2003). The total score equaled the sum of all 25 clinical items. The higher the total score, the more severe any childhood trauma was. The Cronbach’s alpha coefficient in the present study was 0.755.

#### 2.2.2. Chinese Version of Buss & Perry Aggression Questionnaire (AQ-CV)

The Aggression Questionnaire (AQ) is a self-administered inventory that was initially developed by Buss and Perry (1992) and widely used to measure the trait of aggression. It was considered the gold standard for the survey of aggression (Gerevich et al. 2007). The Chinese version of AQ-CV was revised by Li et al. (2011) and is suitable for measuring Chinese college students. The AQ-CV consisted of 30 items which were rated on a five-point Likert scale. Each scale had five forced-choice options ranging from totally disagree to totally agree. The total score was the sum of the numerical answers of 30 items. The higher score of AQ-CV indicated a higher level of aggression. The Cronbach’s alpha coefficient of AQ-CV in the present study was 0.927.

#### 2.2.3. Connor-Davidson Resilience Scale (CD-RISC)

The CD-RISC was originally developed to provide a reliable and valid evaluation of psychological resilience (Connor and Davidson 2003) and was the most widely utilized measurement of resilience (Velickovic et al. 2020). The Chinese version of CD-RISC was translated and back-translated by Yu and Zhang (2007). This scale was rated based on how the participants felt about some particular situation, such as “able to adapt to change,” “coping with stress strengthens,” or “best effort no matter what”. The CD-RISC included 25 items which were rated on a five-point Likert scale. Each item had five forced-choice options ranging from Not true at all to True all the time. The total score was computed by adding all the numerical answers of 25 items. The higher the score of CD-RISC, the greater the psychological resilience. The Cronbach’s alpha coefficient of CD-RISC in the present study was 0.964.

#### 2.2.4. Malevolent Creativity Behavior Scale (MCBS)

The MCBS was utilized to measure the malevolent creative behaviors that occurred in daily life (Hao et al. 2016). Previous studies widely used MCBS to measure individuals’ malevolent creativity (Hao et al. 2020; Jia et al. 2020). This scale consisted of 13 self-assessment items. Instructions for the MCBS asked participants to rate the frequency of the ideas or behaviors of malevolent creativity, such as the idea about “the new ways to punish people”, “how to suppress people who are in your way”, or “how to pull pranks on others”, on a five-point Likert scale with five response options ranging from Never to Always (Hao et al. 2016). The total score of MCBS equaled the sum of all 13 items. The higher score of MCBS indicated more behaviors of malevolent creativity. The Cronbach’s alpha coefficient of MCBS in the present study was 0.883.

### 2.3. Statistics Analysis

The descriptive statistical analysis and correlation analysis were conducted using SPSS. Model 7 in PROCESS 3.3 (Hayes 2013), a macro developed to analyze the mediation and moderation models, was utilized to test the hypothetical moderated mediation model. In model 7, in which psychological resilience was considered as the moderation variable, the interaction role of childhood trauma × psychological resilience predicted the mediating variable (aggressive behavior). The demographic variables of participants containing gender, age, place of birth, and only child status, were entered in the model as covariates. The bias-corrected bootstrap method was used to calculate the 95% confidence interval (CI) for verifying the path coefficient. The effect would be regarded as statistical significance if zero was not contained in the 95% CI.

## 3. Results

### 3.1. Common Method Bias Assessment

Harman’s single-factor test in SPSS was used to assess the common method bias. All the items of CTQ-SF, AQ-CV, CD-RISC, and MCBS were put into the un-rotated exploratory factor analysis. The results showed 19 components with initial eigenvalues greater than one were extracted. The first component accounted for 17.88% of the total variance, which was not greater than the critical value of 40%. The results indicated that the common method bias was not severe in the present sturdy.

### 3.2. Descriptive Statistical Analysis

Table 1 shows the results of the descriptive statistical analysis. The value of the Skewness and Kurtosis showed that the score of CTQ-SF, AQ-CV, CD-RISC, and MCBS basically fitted the normal distribution (Hancock et al. 2010). On the advice from Tabachnick and Fidell (2007) and the large sample size in the present study, the raw data were used for the following statistical analysis.

### 3.3. Correlation Analysis

Table 2 shows the results of the Spearman correlation analysis of the study variable. Results displayed that childhood trauma was significantly associated positively with aggression and malevolent creativity and significantly associated negatively with psychological resilience. The aggression was positively related to malevolent creativity.

### 3.4. Aggression as the Mediator

**Figure 2:**
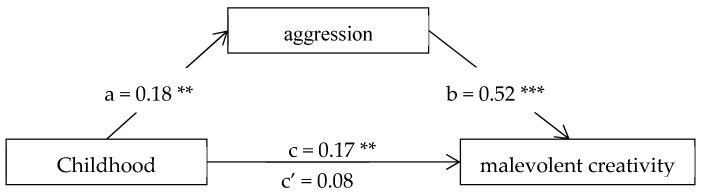
The mediation role of aggression between childhood trauma and malevolent creativity. Note. The mediation model was adjusted for the effects of gender, age, place of birth, and only child status. The letters a, b, c and c’ denote standardized regression coefficients: c = total effect of childhood trauma on malevolent creativity; c’ = direct effect of childhood trauma on malevolent creativity. ** *p* < 0.01, *** *p* < 0.001.

After controlling the effects of gender, age, place of birth, and only child status, the mediation role of aggression between childhood trauma and malevolent creativity was tested using the linear regression analysis based on SPSS. Figure 2 shows the results of multiple linear regression analysis. The total effect (path c) of childhood trauma on malevolent creativity was statistically significant (*c* = 0.17, *p* < 0.01). Both Path *a* (*a* = 0.18, *p* < 0.01) of childhood trauma on aggression and path *b* (*b* = 0.52, *p* < 0.001) of aggression on malevolent creativity were significant. The indirect effect of aggression between childhood trauma and malevolence was 0.09 (a × b), and the 95% CI was 0.038 to 0.159, which indicated that the mediation role of aggression was statistically significant. The ratio of indirect effect to total effect was 52.94%. Additionally, the direct effect (path c’) of childhood trauma on malevolent creativity was marginally significant (*c’* = 0.08, *p* = 0.07), which indicated that the relationship between childhood trauma and malevolent creativity was partially mediated by aggression.

### 3.5. The Moderated Mediation Model Analysis

Model 7 in PROCESS 3.3 developed by (Hayes 2013) was used to explore the hypothetical moderated mediation model in which the indirect relations between childhood trauma and malevolent creativity would be moderated by psychological resilience. The results can be found in Table 3. The results showed that the interaction of childhood trauma and resilience significantly predicted aggression (β = 0.28, *t* = 6.09, *p* < 0.001). This result indicated that psychological resilience moderated the relationship between childhood trauma and aggression.

**Figure 3:**
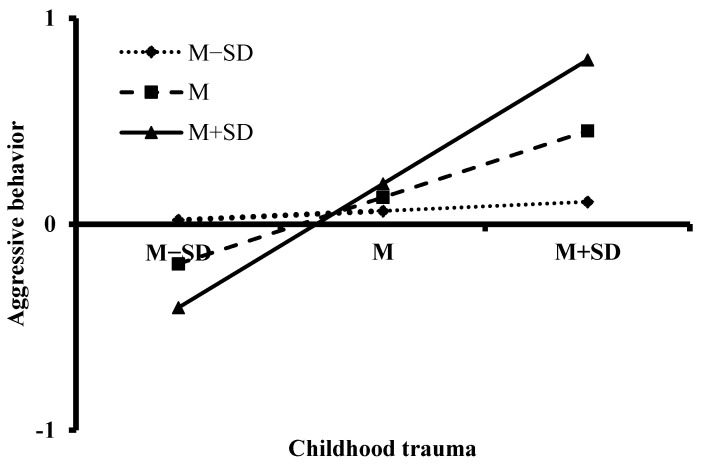
The interaction effect of childhood trauma and psychological resilience on aggression.

Additionally, the simple slope analysis was performed to test the interaction and explore whether the slopes for participants with stronger psychological resilience were different from that for the participants with weaker psychological resilience. The results are shown in Figure 3. The effect of childhood trauma on aggression was stronger for participants with higher (*M* + 1*SD*) psychological resilience (β = 0.60, *t* = 7.59, *p* < 0.001, 95% CI [0.446, 0.757]) than that for participants with medium (*M*) psychological resilience (β = 0.32, *t* = 5.76, *p* < 0.001, 95% CI [0.213, 0.433]) and lower (*M* − 1*SD*) psychological resilience (β = 0.04, *t* = 0.68, *p* = 0.495, 95% CI [−0.083, 0.172]). That is to say, the aggressive behaviors of college students with high and medium levels of psychological resilience were more likely to be affected by childhood trauma than that of college students with a low level of psychological resilience. Meanwhile, resilience buffered the negative roles of low-level childhood trauma on aggression but magnified the negative effects of high-level childhood trauma.

Further, the moderated mediation model analysis revealed that the mediation role of aggression between childhood trauma and malevolent creativity was statistically moderated by psychological resilience (Index = 0.14, *SE* = 0.03, 95% CI [0.092, 0.205]). The results are shown in Table 4, indicating that the conditional indirect effect was 0.02, 95% CI (−0.047, 0.090) for −1SD resilience, 0.17, 95% CI (0.102, 0.243) for M resilience, and 0.31, 95% CI (0.213, 0.432) for +SD resilience. Thus, the moderated mediation assumption regarding psychological resilience was fully supported.

## 4. Discussion

In the present study, a moderated mediation model was used to investigate the mechanisms underlying the association between earlier childhood trauma and later malevolent creativity. Our findings indicated that the correlation between childhood trauma and malevolent creativity is mediated by aggression. Exposure to more childhood trauma increased aggressive behavior, which in turn boosted the likelihood of malevolent creativity. Moreover, this indirect connection was moderated by psychological resilience in that the indirect effects of childhood trauma on malevolent creativity through aggression were only significant in participants with high resilience and not those with low resilience.

### 4.1. The Association between Childhood Trauma and Malevolent Creativity

The results show that childhood trauma was positively correlated with malevolent creativity. This implies that individuals who experienced more trauma in childhood were more likely to generate more malevolent creative behaviors in adulthood. Similarly, a survey investigated in China also showed that childhood neglect was positively associated with malevolent creativity (Jia et al. 2020). Other studies also found that parental negligence facilitated children’s antisocial behavior and decreased their pro-social behavior (Llorca et al. 2017). Additionally, Guo et al. (2021) found that parental warmth was positively correlated with benevolent creativity. The present results underlines that the association between home environment and individual creativity cultivation is extremely complicated. That is, an advantageous home environment, parenting attitudes, and growing-up experiences facilitate the development of benevolent creativity behavior, while disadvantaged ones not only prevent its development but promote the development of malevolent creativity behavior (Guo et al. 2021). In the light of social information processing theory, people who have been exposed to more neglect and abuse in early life may be more likely to consider neutral social information as threatening cues, which could evoke hostile thinking (Gawronski and Cesario 2013) and aggressive behavior (Mobbs et al. 2015). Furthermore, traumatized individuals tend to be more vulnerable and easily get anxious or depressed when exposed to a threatening environment (Infurna et al. 2016; Zhao et al. 2022b). From the perspective of developmental psychology, emotion is an ability that emerges from various properties containing attention, memory, theory of mind, and categorization. Each of these abilities may be affected by childhood maltreatment (Ruba and Pollak 2020). What’s more, individuals immersed in negative emotions are more likely to be more introspective, analytical, and insistent on their inner cognitive processing (De Dreu et al. 2012). Therefore, they can generate more original, useful, and harmful ways to harm others (Jia et al. 2020).

### 4.2. The Mediating Role of Aggression

The findings that aggression partially mediated the connection between childhood trauma and malevolent creativity are consistent with Hypothesis 2. The results indicated that aggression was positively associated with malevolent creativity, which implies that individuals with a high level of aggression usually show more malevolent creativity behaviors. This is consistent with the study by Lee and Dow (2011), who reported that the trait of physical aggression is positively associated with malevolent creativity as measured by divergent thinking tasks. Other studies also report that malevolently creative ideas generated in the problem-solving task were significantly greater in participants who are more implicitly aggressive than in participants who are less implicitly aggressive. In addition, our findings indicated that childhood trauma was positively associated with aggression. This result implies that individuals who are exposed to more maltreatment in early life usually show more aggressive behaviors in adolescence or adulthood. This is consistent with vast numbers of previous studies (He and Xiang 2021; Ma et al. 2022; Schwarzer et al. 2021; Xiao et al. 2021), which showed that maltreated individuals are more likely to generate aggressive behavior. This result is also consistent with the theoretical perspectives of GAM (Anderson and Bushman 2002), which indicated that individuals exposed to childhood trauma tend to normalize the use of violence and increase aggressive behavior. Therefore, individuals who were abused or neglected in childhood are more likely to generate more aggressive behaviors and then generate more malevolent creativity behaviors.

### 4.3. The Moderating Role of Psychological Resilience

This study further found that psychological resilience moderated the association between childhood trauma and aggression. The present results indicated that individuals with high resilience are more likely to be aggressive when they have experienced abuse and neglect in childhood. In other words, individuals exposed to high levels of childhood trauma were more likely to engage in aggressive behaviors when the level of resilience was high, while individuals exposed to low levels of childhood trauma were more likely to engage in aggressive behaviors when the level of resilience was low. There is inconsistent evidence concerning the boon of resilience for individuals who have experienced adversity. Some studies indicated that high-resilience individuals have enough ability to adapt to stressors or adversity and maintain mental health in the presence of stressful events or adversity (Kalisch et al. 2015; VanMeter and Cicchetti 2020). Other studies also showed that psychological resilience could attenuate the aggressive behaviors in individuals who had been exposed to early life stress (Kim et al. 2015; Nooripour et al. 2022).

One possible explanation might correlate with the stress-buffering model and reverse the stress-buffering model, which provides the theoretical framework with regard to the moderating effect of social support on the association between stressful events and depression (Rueger et al. 2016). The stress-buffering model posits that the negative effects of stress on mental health are more serious among those with insufficient social support than those with sufficient support (Rueger et al. 2016). Our results showed that the deleterious effects of low-level childhood trauma are greater among those with limited psychological resilience than those with adequate resilience. However, the reverse stress-buffering model supposes that the negative effects of stress on mental health are greater among those with sufficient support than those with insufficient support (Rueger et al. 2016). Our results were consistent with the reverse stress-buffering model. The harmful effects of high-level childhood trauma are greater among those with adequate resilience than those with limited resilience. Therefore, these results indicate that childhood trauma with different severity may not allow the individual to fully utilize the benefits of psychological resilience. It is possible to assume that some early traumatic contexts may restrain the effects of resilience, which emphasize the potential advantage of both “stress-buffering” (effects of resilience are enhanced while the level of childhood trauma is low) and “reverse stress-buffering” (effects of resilience are dampened while the level of childhood trauma is high).

Another possible explanation could be that resilience is a dynamic and ordinary process instead of a rare and extraordinary process (Masten 2001). Rutter (2012) pointed out that resilience has a “huge heterogeneity in response to all manners of environmental hazards”. Fincham et al. (2009) found, for example, that an individual’s resilience could not buffer the negative roles of high levels of stress on post-traumatic stress disorder (PTSD) symptoms. In their review article on re-evaluating resilience across various levels of risk, Vanderbilt-Adriance and Shaw (2008) indicated that psychological resilience might not attenuate the negative effects of childhood adversity because they were at a too high level of stress. Pauly et al. (2021) also found that a high-level conscientious persona trait correlated with high-level stress in individuals with high-level resilience. These studies seemed to indicate that the buffer effect of resilience was conditional on the severity of the negative or adverse experiences. Additionally, high levels of positive emotions were typically characteristic of psychological resilience (Tugade and Fredrickson 2004). Another possible reason could be that high levels of childhood trauma and high levels of psychological resilience could be correlated with a strong feeling of having to release tension, stress, and pain, which might derive from childhood abuse and neglect. In turn, this would cause further aggressive behaviors. Thus, future studies will contribute to determining more consistent associations among childhood trauma, aggression, and psychological resilience. Particularly, the moderated mediation analysis revealed that psychological resilience moderated the strength of the association between childhood trauma and malevolent creativity mediated by aggression. That is, the indirect effect was highest in the group with high psychological resilience while lowest with those in the low resilient group. It seems that with increasing psychological resilience, the correlation between childhood trauma and malevolent creativity mediated by aggression was strengthened. However, when resilience decreased to a certain level (lower than *M* − *SD*), the indirect effect of childhood trauma on malevolent creativity had no statistical significance. This results partially consistented with the association between psychological resilience and creativity (Anser et al. 2022; Chen 2015; Marwa and Milner 2013; Xu et al. 2021), which indicated that psychological resilience positively predicted benevolent creativity. Thus, psychological resilience might also increase the emergence of malevolent creativity when individuals exposed to childhood trauma. Additionally, it is also possible to speculate that the buffering effects of resilience are enhanced while the level of childhood trauma is low, and the effects of resilience are dampened while the level of childhood trauma is high.

This finding is roughly consistent with the perspectives of Rutter (2012), who claimed that exposure to adversity might contribute to increased resilience to later adversity (called the steeling effect) rather than a sensitization or increased vulnerability. In the present study, the association between severe trauma and adversity with increased aggressive behaviors and malicious creative behaviors may reflect the steeling effect (Rutter 2012), which released negative effects of childhood trauma by attacking others and further resulted in more malevolent creativity. The steeling effects might let individuals with greater childhood abuse and/or neglect be better able to successfully adapt to adversity or stressors by the emergence of aggressive behaviors instead of depression and maintain mental health in the face of trauma or stressor. Of course, further studies are needed to explore this conjecture of whether individuals with low-level resilience may be more vulnerable to depression when exposed to high-level childhood trauma, and individuals with high-level resilience may display more aggressive behaviors instead of depressive symptoms.

## 5. Limitations and Implications

There are some limitations of the present research that should be mentioned. Firstly, the cross-sectional design and correlation analysis used in the present study to investigate the moderated mediation model cannot establish the causal association among study variables and may lead to possible biased estimates of parameters (Maxwell and Cole 2007). Further longitudinal or experimental studies will be needed to identify the moderated mediation model. Secondly, the retrospective self-reported questionnaire was used to measure childhood trauma, which may lead to inaccurate answers because of the distant memory that occurred in childhood. Multiple forms of measures should be taken to assess early life experiences. Thirdly, the participants enrolled in the present study were all undergraduates, who are a special group compared with other age colonies. This limited the interpretation and prediction of the malevolent creativity of other age colonies based on the results of the present study. Various age groups will be needed to replicate our findings in future studies. Only then will it be possible to improve the quality of the survey data and provide more possible insights into the associations among variables investigated than that being explored in the present study.

Although the present study has some limitations, these should not overshadow its implications. The results of the present study further explain malevolent creativity in connection with early environmental and individual difference factors and extend the understanding of malevolent creativity. Childhood adverse experiences can strongly facilitate the emergence of malevolent creativity. These negative growing environments also have indirect effects on malevolent creativity through the mediating role of aggression, but depending on a person’s resilience level. With that in mind, the interaction effect indicated that high resilience could buffer the negative roles of low-level childhood trauma on aggression but magnify the negative effects of high-level childhood trauma. Resilient individuals with exposure to high-level childhood trauma will have more aggressive behavior. Therefore, we must draw attention to the malevolent creativity of individuals who have experienced more childhood abuse and neglect and have high-level resilience and guide them with the proper way to due attention to childhood trauma and decrease the expression of malevolent creativity.

## 6. Conclusions

In summary, the present study further validated the association between childhood trauma and malevolent creativity in the sample of Chinese college students. The findings illustrated the mediating effect of aggression in the pathway from childhood trauma to malevolent creativity behavior. Additionally, the results also showed evidence of two-way interaction, indicating that psychological resilience moderated the correlation between childhood trauma and aggression. Participants with low resilience had bigger changes of aggression and smaller changes of malevolent creativity across both low and high levels of childhood trauma than those with high resilience.

## Figures and Tables

**Table 1:** Descriptive statistical results of study variables.

Variables	M	SD	Skewness	Kurtosis
Childhood trauma	36.70	10.62	0.90	−0.15
Aggression	56.70	16.47	0.29	−0.25
Psychological resilience	80.64	21.56	−0.47	0.19
Malevolent creativity	9.30	7.00	0.67	−0.22

**Table 2:** Correlation analysis results of study variables.

	1	2	3	4
1. Childhood trauma	—			
2. Aggression	0.23 ***	—		
3. Psychological resilience	−0.45 ***	−0.07	—	
4. Malevolent creativity	0.19 ***	0.56 ***	0.01	—

Note: *** *p* < 0.001.

**Table 3:** Testing the moderation effect of resilience.

	Aggression			
	β	*SE*	*t*	95% CI
Gender	0.09	0.050	1.83	−0.007, 0.190
Age	0.04	0.049	0.77	−0.058. 0.133
Residence	0.03	0.051	0.59	−0.071, 0.131
One child	0.03	0.052	0.64	−0.069, 0.135
Childhood trauma	0.32	0.056	5.76 ***	0.213, 0.433
Resilience	0.07	0.056	1.18	−0.044, 0.176
Childhood trauma × resilience	0.28	0.046	6.09 ***	0.189, 0.369
*R* ^2^	0.15			
*F*	9.62 ***			

Note. *SE*, standard error. CI, confidence interval. *** *p* < 0.001.

**Table 4:** The moderated indirect effect.

Psychological Resilience	Indirect Effect	BootSE	95% CI
*M* − *SD*	0.02	0.035	−0.047, 0.090
*M*	0.17	0.036	0.102, 0.243
*M* + *SD*	0.31	0.056	0.213, 0.432

## Data Availability

The data is available on request to the corresponding author.

## References

[B1-jintelligence-10-00097] Acland Erinn L., Jambon Marc, Malti Tina (2021). Children’s emotion recognition and aggression: A multi-cohort longitudinal study. Aggressive Behavior.

[B2-jintelligence-10-00097] Anderson Craig A., Bushman Brad J. (2002). Human Aggression. Annual Review of Psychology.

[B3-jintelligence-10-00097] Anser Muhammad Khalid, Zahid Yousaf, Muhammad Sharif, Wang Yijun, Abdu Majid, Muhammad Yasir (2022). Investigating Employee Creativity Through Employee Polychronicity and Employee Resilience: A Glimpse of Nurses Working in the Health-Care Sector. European Journal of Innovation Management.

[B4-jintelligence-10-00097] Anyan Frederick, Hjemdal Odin (2016). Adolescent stress and symptoms of anxiety and depression: Resilience explains and differentiates the relationships. Journal of Affective Disorders.

[B5-jintelligence-10-00097] Baas Matthijs, Roskes Marieke, Koch Severine, Cheng Yujie, De Dreu Carsten K. W. (2019). Why Social Threat Motivates Malevolent Creativity. Personality and Social Psychology Bulletin.

[B6-jintelligence-10-00097] Beaussart Melanie L., Andrews Candace J., Kaufman James C. (2013). Creative liars: The relationship between creativity and integrity. Thinking Skills and Creativity.

[B7-jintelligence-10-00097] Bernardes Elisa Teixeira, Manitto Alicia Matijasevich, Miguel Eurípedes Constantino, Pan Pedro Mario, Batistuzzo Marcelo Camargo, Rohde Luis Augusto, Polanczyk Guilherme V. (2020). Relationships between childhood maltreatment, impairment in executive functions and disruptive behavior disorders in a community sample of children. European Child and Adolescent Psychiatry.

[B8-jintelligence-10-00097] Bernstein David P., Stein Judith A., Newcomb Michael D, Walker Edward, Pogge David, Ahluvalia Taruna, Zule William (2003). Development and validation of a brief screening version of the Childhood Trauma Questionnaire. Child Abuse and Neglect.

[B9-jintelligence-10-00097] Bernstein David P., Fink Laura, Handelsman Leonard, Foote Jeffrey, Lovejoy Meg, Wenzel Katherine, Ruggiero Joseph (1994). Initial reliability and validity of a new retrospective measure of child abuse and neglect. The American Journal of Psychiatry.

[B10-jintelligence-10-00097] Bland Vikki J., Lambie Ian, Best Charlotte (2018). Does childhood neglect contribute to violent behavior in adulthood? A review of possible links. Clinical Psychology Review.

[B11-jintelligence-10-00097] Buss Arnold H., Perry Mark (1992). The aggression questionnaire. Journal of Personality and Social Psychology.

[B12-jintelligence-10-00097] Canale Natale, Marino Claudia, Griffiths Mark Damian, Scacchi Luca, Monaci Maria Grazia, Vieno Alessio (2019). The association between problematic online gaming and perceived stress: The moderating effect of psychological resilience. Journal of Behavioral Addictions.

[B13-jintelligence-10-00097] Chang Junjie, Ji Yan, Li Yonghan, Yuan Mengyuan, Su Puyu (2021). Childhood trauma and depression in college students: Mediating and moderating effects of psychological resilience. Asian Journal of Psychiatry.

[B14-jintelligence-10-00097] Chen Mei-Fang (2015). An examination of the value-belief-norm theory model in predicting pro-environmental behaviour in Taiwan. Asian Journal of Social Psychology.

[B15-jintelligence-10-00097] Chen Xinyin, Huang Xiaorui, Chang Lei, Wang Li, Li Dan (2010). Aggression, social competence, and academic achievement in Chinese children: A 5-year longitudinal study. Development and Psychopathology.

[B16-jintelligence-10-00097] Chiasson Carley, Moorman Jessie, Romano Elisa, Vezarov Michel, Cameron Andrew, Smith Andra (2021). The influence of emotion on working memory: Exploratory fMRI findings among men with histories of childhood sexual abuse. Child Abuse & Neglect.

[B17-jintelligence-10-00097] Clark Karla, James Keith (1999). Justice and Positive and Negative Creativity. Creativity Research Journal.

[B18-jintelligence-10-00097] Cohen Jacob (1992). Statistical Power Analysis. Current Directions in Psychological Science.

[B19-jintelligence-10-00097] Connor Kathryn M., Davidson Jonathan R. T. (2003). Development of a new resilience scale: The Connor-Davidson Resilience Scale (CD-RISC). Depression and Anxiety.

[B20-jintelligence-10-00097] Cropley Arthur, Cropley David (2011). Creativity and Lawbreaking. Creativity Research Journal.

[B21-jintelligence-10-00097] Cropley David H., Kaufman James C., Cropley Arthur J. (2008). Malevolent Creativity: A Functional Model of Creativity in Terrorism and Crime. Creativity Research Journal.

[B22-jintelligence-10-00097] Cropley David H., Kaufman James C., White Arielle E., Chiera Belinda A. (2014). Layperson perceptions of malevolent creativity: The good, the bad, and the ambiguous. Psychology of Aesthetics, Creativity, and the Arts.

[B23-jintelligence-10-00097] De Beilis Michael D., Putnam Frank W. (1994). The Psychobiology of Childhood Maltreatment. Child and Adolescent Psychiatric Clinics of North America.

[B24-jintelligence-10-00097] De Dreu Carsten K., Nijstad Bernard A., Baas Matthijs, Wolsink Inge, Roskes Marieke (2012). Working memory benefits creative insight, musical improvisation, and original ideation through maintained task-focused attention. Personality & Social Psychology Bulletin.

[B25-jintelligence-10-00097] Doba Karyn, Saloppé Xavier, Choukri Fatima, Nandrino Jean-Louis (2022). Childhood trauma and posttraumatic stress symptoms in adolescents and young adults: The mediating role of mentalizing and emotion regulation strategies. Child Abuse & Neglect.

[B26-jintelligence-10-00097] Fava Nicole M., Trucco Lisa M., Martz Meghan E., Cope Lora M., Jester Jennifer M., Zucker Robert A., Heitzeg Mary M. (2019). Childhood adversity, externalizing behavior, and substance use in adolescence: Mediating effects of anterior cingulate cortex activation during inhibitory errors. Development and Psychopathology.

[B27-jintelligence-10-00097] Fedina Lisa, Nam Boyoung, Jun Hyun-Jin, Shah Roma, Von Mach Tara, Bright Charlotte L., DeVylder Jordan (2021). Moderating Effects of Resilience on Depression, Psychological Distress, and Suicidal Ideation Associated with Interpersonal Violence. The Journal of Interpersonal Violence.

[B28-jintelligence-10-00097] Fincham Dylan S., Altes Lucas Korthals, Stein Dan J., Seedat Soraya (2009). Posttraumatic stress disorder symptoms in adolescents: Risk factors versus resilience moderation. Comprehensive Psychiatry.

[B29-jintelligence-10-00097] Fletcher Jason M., Schurer Stefanie (2017). Origins of Adulthood Personality: The Role of Adverse Childhood Experiences. The B.E. Journal of Economic Analysis & Policy.

[B30-jintelligence-10-00097] Gawronski Bertram, Cesario Joseph (2013). Of Mice and Men:What Animal Research Can Tell Us About Context Effects on Automatic Responses in Humans. Personality and Social Psychology Review.

[B31-jintelligence-10-00097] Georgieva Sylvia, Tomas Jose Manuel, Navarro-Pérez Javier Jose (2021). Systematic review and critical appraisal of Childhood Trauma Questionnaire—Short Form (CTQ-SF). Child Abuse and Neglect.

[B32-jintelligence-10-00097] Gerevich Joesf, Bácskai Erika, Czobor Pal (2007). The generalizability of the Buss-Perry Aggression Questionnaire. International Journal of Methods in Psychiatric Research.

[B33-jintelligence-10-00097] Gill Paul, Horgan John, Hunter Samuel T., Cushenbery Lily D. (2013). Malevolent Creativity in Terrorist Organizations. The Journal of Creative Behavior.

[B34-jintelligence-10-00097] Gong Jingbo, Liu Jianbo, Shangguan Lizhi, Zhang Qin, Peng Zhu, Li Zun, Shi Lijuan (2019). Childhood maltreatment impacts the early stage of facial emotion processing in young adults with negative schizotypy. Neuropsychologia.

[B35-jintelligence-10-00097] Gong Zhe, Liu Chang (2016). Malevolent Creativity: Concept, Measurement, Influence Factors and Future Research. Journal of Psychological Science.

[B36-jintelligence-10-00097] Guo Jiajun, Zhang Jing, Pang Weiguo (2021). Parental warmth, rejection, and creativity: The mediating roles of openness and dark personality traits. Personality and Individual Differences.

[B37-jintelligence-10-00097] Gutworth Melissa B., Cushenbery Lily, Hunter Samuel T. (2018). Creativity for Deliberate Harm: Malevolent Creativity and Social Information Processing Theory. The Journal of Creative Behavior.

[B38-jintelligence-10-00097] Hancock Gregory R., Stapleton Laura M., Mueller Ralph O. (2010). The Reviewer’s Guide to Quantitative Methods in the Social Sciences.

[B39-jintelligence-10-00097] Hao Ning, Tang Mengying, Yang Jing, Wang Qifei, Runco Mark A. (2016). A New Tool to Measure Malevolent Creativity: The Malevolent Creativity Behavior Scale. Frontiers in Psychology.

[B40-jintelligence-10-00097] Hao Ning, Qiao Xinuo, Cheng Rui, Lu Kelong, Tang Mengying, Runco Mark A. (2020). Approach motivational orientation enhances malevolent creativity. Acta Psychologica.

[B41-jintelligence-10-00097] Harris Daniel J., Reiter-Palmon Roni (2015). Fast and furious: The influence of implicit aggression, premeditation, and provoking situations on malevolent creativity. Psychology of Aesthetics, Creativity, and the Arts.

[B42-jintelligence-10-00097] Harris Daniel J., Reiter-Palmon Roni, Kaufman James C. (2013). The effect of emotional intelligence and task type on malevolent creativity. Psychology of Aesthetics, Creativity, and the Arts.

[B43-jintelligence-10-00097] Hayes Andrew F. (2013). Introduction to Mediation, Moderation, and Conditional Process Analysis: A Regression-Based Approach.

[B44-jintelligence-10-00097] He Nina, Xiang Yanhui (2021). How child maltreatment impacts internalized/externalized aggression among Chinese adolescents from perspectives of social comparison and the general aggression model. Child Abuse & Neglect.

[B45-jintelligence-10-00097] Infurna Maria Rita, Reichl Corinna, Parzer Peter, Schimmenti Adriano, Bifulco Antonia, Kaess Michael (2016). Associations between depression and specific childhood experiences of abuse and neglect: A meta-analysis. Journal of Affective Disorders.

[B46-jintelligence-10-00097] James Keith, Clark Karla, Cropanzano Russell (1999). Positive and negative creativity in groups, institutions, and organizations: A model and theoretical extension. Creativity Research Journal.

[B47-jintelligence-10-00097] Jia Xuji, Wang Qingjin, Lin Lin (2020). The Relationship Between Childhood Neglect and Malevolent Creativity: The Mediating Effect of the Dark Triad Personality. Frontiers in Psychology.

[B48-jintelligence-10-00097] Jonason Peter K., Abboud Rookaya, Tomé Jordi, Dummett Melanie, Hazer Ashleigh (2017). The Dark Triad traits and individual differences in self-reported and other-rated creativity. Personality and Individual Differences.

[B49-jintelligence-10-00097] Kalisch Raffael, Müller Marianne B, Tüscher Oliver (2015). A conceptual framework for the neurobiological study of resilience. Behavioral and Brain Sciences.

[B50-jintelligence-10-00097] Kim Joohan, Seok Jeong-Ho, Choi Kang, Jon Duk-In, Hong Hyun Ju, Hong Narei, Lee Eunjeong (2015). The Protective Role of Resilience in Attenuating Emotional Distress and Aggression Associated with Early-life Stress in Young Enlisted Military Service Candidates. Journal of Korean Medical Science.

[B51-jintelligence-10-00097] Lawrence-Wood Ellie, Baur Jenelle, Lawrence Andrew, Forbes David, McFarlane Alexander (2021). The role of inhibitory processes in the relationship between subsyndromal PTSD symptoms and aggressive behaviour. Journal of Psychiatric Research.

[B52-jintelligence-10-00097] Lee Sherman A., Dow Gayle T. (2011). Malevolent Creativity: Does Personality Influence Malicious Divergent Thinking?. Creativity Research Journal.

[B53-jintelligence-10-00097] Li Xianyun, Phillips Michael R., Zhang Yali, Niu Yajuan, Tong Yongshen, Yang Shaojie (2011). Development, Reliability and Validity of the Chinese version of Buss & Perry Aggression Questionnaire. Chinese Journal of Nervous and Mental Diseases.

[B54-jintelligence-10-00097] Llorca Anna, Richaud María Cristina, Malonda Elisabeth (2017). Parenting Styles, Prosocial, and Aggressive Behavior: The Role of Emotions in Offender and Non-offender Adolescents. Frontiers in Psychology.

[B55-jintelligence-10-00097] Logan-Greene Patricia, Semanchin Jones Annette (2015). Chronic neglect and aggression/delinquency: A longitudinal examination. Child Abuse & Neglect.

[B56-jintelligence-10-00097] Ma Julie, Han Yoonsun, Kang Hae Rin (2022). Physical punishment, physical abuse, and child behavior problems in South Korea. Child Abuse & Neglect.

[B57-jintelligence-10-00097] Marwa Simmy M., Milner Christopher D. (2013). Underwriting corporate resilience via creativity: The pliability model. Total Quality Management & Business Excellence.

[B58-jintelligence-10-00097] Masten Ann S. (2001). Ordinary magic. Resilience processes in development. American Psychologist.

[B59-jintelligence-10-00097] Maxwell Scott E., Cole David A. (2007). Bias in cross-sectional analyses of longitudinal mediation. Psychological Methods.

[B60-jintelligence-10-00097] Min Jung-Ah, Lee Chang-Uk, Chae Jeong-Ho (2015). Resilience moderates the risk of depression and anxiety symptoms on suicidal ideation in patients with depression and/or anxiety disorders. Comprehensive Psychiatry.

[B61-jintelligence-10-00097] Mobbs Dean, Hagan Cindy C., Dalgleish Tim, Silston Brian, Prévost Charlotte (2015). The ecology of human fear: Survival optimization and the nervous system [Hypothesis and Theory]. Frontiers in Neuroscience.

[B62-jintelligence-10-00097] Nooripour Roghieh, Hoseinian Simin, Vakili Yaghoob, Ghanbari Nikzad, Matacotta Joshua J., Mozaffari Nazir, Lavie Carl (2022). Psychometric properties of Farsi version of the resilience scale (CD-RISC) and its role in predicting aggression among Iranian athletic adolescent girls. BMC Psychology.

[B63-jintelligence-10-00097] Odgers Candice L., Moffitt Terrie E., Broadbent Jonathan M., Dickson Nigel, Hancox Robert J., Harrington Honalee, Caspi Avshalom (2008). Female and male antisocial trajectories: From childhood origins to adult outcomes. Development and Psychopathology.

[B64-jintelligence-10-00097] Pauly Claire, Ribeiro Fabiana, Schröder Valerie E., Pauly Laure, Krüger Rejko, Leist Ania K. (2021). The Moderating Role of Resilience in the Personality-Mental Health Relationship During the COVID-19 Pandemic. Frontiers in Psychiatry.

[B65-jintelligence-10-00097] Perchtold-Stefan Corinna M., Fink Andreas, Rominger Christian, Papousek Ilona (2021). Creative, Antagonistic, and Angry? Exploring the Roots of Malevolent Creativity with a Real-World Idea Generation Task. The Journal of Creative Behavior.

[B66-jintelligence-10-00097] Rasche Katharina, Dudeck Manuela, Otte Stefanie, Klingner Solveig, Vasic Nenad, Streb Judith (2016). Factors influencing the pathway from trauma to aggression: A current review of behavioral studies. Neurology, Psychiatry and Brain Research.

[B67-jintelligence-10-00097] Ruba Ashley L., Pollak Seth D. (2020). Children’s emotion inferences from masked faces: Implications for social interactions during COVID-19. PLoS ONE.

[B68-jintelligence-10-00097] Rueger Sandra Yu, Malecki Christine Kerres, Pyun Yoonsun, Aycock Chase, Coyle Samantha (2016). A meta-analytic review of the association between perceived social support and depression in childhood and adolescence. Psychological Bulletin.

[B69-jintelligence-10-00097] Runco Mark A. (2004). Creativity. Annual Review of Psychology.

[B70-jintelligence-10-00097] Runco Mark A., Jaeger Garrett J. (2012). The Standard Definition of Creativity. Creativity Research Journal.

[B71-jintelligence-10-00097] Rutter M. (2012). Resilience as a dynamic concept. Development and Psychopathology.

[B72-jintelligence-10-00097] Rutter Michael (1999). Resilience concepts and findings: Implications for family therapy. Journal of Family Therapy.

[B73-jintelligence-10-00097] Schwarzer Nicola-Hans, Nolte Tobias, Fonagy Peter, Gingelmaier Stephan (2021). Mentalizing mediates the association between emotional abuse in childhood and potential for aggression in non-clinical adults. Child Abuse & Neglect.

[B74-jintelligence-10-00097] Spann Marisa N., Mayes Linda C., Kalmar Jessica H., Guiney Joanne, Womer Fay Y., Pittman Brian, Blumberg Hilary P. (2012). Childhood abuse and neglect and cognitive flexibility in adolescents. Child Neuropsychology.

[B75-jintelligence-10-00097] Sternberg Robert J., Lubart Todd I., Sternberg R. J. (1999). The concept of creativity: Prospects and paradigms. Handbook of Creativity.

[B76-jintelligence-10-00097] Tabachnick Barbara G., Fidell Linda S. (2007). Using Multivariate Statistics.

[B77-jintelligence-10-00097] Tugade Michele M., Fredrickson Barbara L. (2004). Resilient individuals use positive emotions to bounce back from negative emotional experiences. Journal of Personality and Social Psychology.

[B78-jintelligence-10-00097] Vanderbilt-Adriance Ella, Shaw Daniel S. (2008). Conceptualizing and Re-Evaluating Resilience Across Levels of Risk, Time, and Domains of Competence. Clinical Child and Family Psychology Review.

[B79-jintelligence-10-00097] VanMeter Faith, Cicchetti Dante (2020). Resilience. Handbook of Clinical Neurology.

[B80-jintelligence-10-00097] Velickovic Katarina, Hallberg Ingalill Rahm, Axelsson Ulrika, Borrebaeck Carl A. K., Rydén Lisa, Johnsson Per, Månsson Johanna (2020). Psychometric properties of the Connor-Davidson Resilience Scale (CD-RISC) in a non-clinical population in Sweden. Health Quality of Life Outcomes.

[B81-jintelligence-10-00097] Vuoksimaa Eero, Rose Richard J., Pulkkinen Lea, Palviainen Teemu, Rimfeld Kaili, Lundström Sebastain, Kaprio Jaakko (2021). Higher aggression is related to poorer academic performance in compulsory education. Journal Child Psychology and Psychiatry.

[B82-jintelligence-10-00097] Wattick Rachel A., Olfert Melissa D. (2022). P026 Adverse Childhood Experiences and Social Support of Young Adults with Food Addiction. Journal of Nutrition Education and Behavior.

[B83-jintelligence-10-00097] Werner Earl E. (1996). Vulnerable but invincible: High risk children from birth to adulthood. European Child and Adolescent Psychiatry.

[B84-jintelligence-10-00097] Xiao Zhuoni, Baldwin Mina Murat, Meinck Franziska, Obsuth Ingrid, Murray Aja Louise (2021). The impact of childhood psychological maltreatment on mental health outcomes in adulthood: A protocol for a systematic review and meta-analysis. Systematic Reviews.

[B85-jintelligence-10-00097] Xu Yanhua, Shao Jinlian, Zeng Wei, Wu Xingrou, Huang Dongtao, Zeng Yuqing, Wu Jiamin (2021). Depression and Creativity During COVID-19: Psychological Resilience as a Mediator and Deliberate Rumination as a Moderator. Frontiers in Psychology.

[B86-jintelligence-10-00097] Yu Xiaonan, Zhang Jianxin (2007). Factor analysis and psychometric evaluation of the Connor-Davidson Resilience Scale (CD-RISC) with Chinese people. Social Behavior and Personality.

[B87-jintelligence-10-00097] Zhang Dongjing, Zhou Zongkui, Gu Chuanhua, Lei Yuju, Fan Cuiying (2018). Family Socio-Economic Status and Parent-Child Relationships Are Associated with the Social Creativity of Elementary School Children: The Mediating Role of Personality Traits. Journal of Child and Family Studies.

[B88-jintelligence-10-00097] Zhao Jingwen, Xu Xiaobo, Pang Weiguo (2022a). When do creative people engage in malevolent behaviors? The moderating role of moral reasoning. Personality and Individual Differences.

[B89-jintelligence-10-00097] Zhao Xingfu, Zhang Yalin, Li Longfei, Zhou Yunfei, Li Hezhan, Yang Shichang (2005). Reliability and validity of the Chinese version of childhood trauma questionnaire. Chinese Journal of Clinical Rehabilitation.

[B90-jintelligence-10-00097] Zhao Yuxiao, Han Lin, Teopiz Kayla M., McIntyre Roger S., Ma Ruining, Cao Bing (2022b). The psychological factors mediating/moderating the association between childhood adversity and depression: A systematic review. Neuroscience & Biobehavioral Reviews.

